# COVID-19 and the Nigerian correctional service: need for structured data

**DOI:** 10.11604/pamj.supp.2020.37.1.25370

**Published:** 2020-09-28

**Authors:** Asma Qureshi, Ima Kashim, Similolu Akintorin, Larissa Unruh, Sadhana Dharmapuri, Kenneth Soyemi

**Affiliations:** 1 Department of Trauma Burn and Critical Care, Cook County Health System, Chicago, Illinois, USA,; 2 Department of Economic and Social Infrastructure, Islamic Development Bank, Chicago, Illinois, USA,; 3 Keck School of Medicine at the University of Southern California, Los Angeles, CA, USA,; 4 Department of Emergency Medicine John H. Stroger Jr. Hospital of Cook County Health, Chicago, Illinois, USA,; 5 Cermark Health Services, Cook County Juvenile Temporary Detention Center, Chicago, Illinois, USA,; 6 Department of Pediatrics, John H. Stroger Jr. Hospital of Cook County, Chicago, Illinois, USA

**Keywords:** COVID-19, response, prison data, point-of-care testing, intervention, non-pharmaceutical

## Abstract

Although prisoners are considered a vulnerable population, no data repository currently exists to monitor the COVID-19 incidence in Nigerian prisons. To better understand the impact of COVID-19 within the Nigerian prison system, prisons should develop detailed COVID-19 response protocols, implement enhanced point-of-care testing, and initiate contact tracing with meticulous data collection.

## Brief

The SARS-CoV-2 (COVID-19) pandemic has caused significant worldwide morbidity and mortality [1]. In Nigeria, the first case of COVID-19 was reported on February 27, 2020, and since then approximately 275,000 Real Time Polymerase Chain Reaction (RT-PCR) tests have been reported from the country´s 59 testing laboratories [2]. Testing metrics show: 1) a daily testing rate of 1.0 per 100,000 population, 2) a confirmed case rate of 2.5 per million population, and 3) a positivity rate of 15.3 to 17.6% [3]. A general benchmark for adequate testing is a positivity rate between 3 to 12%, indicating that Nigeria is not testing widely enough or has low testing capacity [4]. An effective pandemic response requires that testing, tracing, and treatment be scaled to the size of the outbreak, not the size of the population. Underreporting of disease in vulnerable populations, including incarcerated individuals, is a worldwide concern. Correctional facilities in the United States, have implemented various mitigation and prevention strategies based on rigorous data reporting. For example, one large metropolitan correctional facility in the United States chose to release low-risk prisoners to home confinement [5,6].

In this report, we propose that prevention efforts within the Nigerian Correctional Service should establish a structured data collection system to enhance understanding of disease spread and guide the design and implementation of evidence-based prevention and control measures [7]. Currently, a total of 71,522 prisoners are incarcerated in 240 locations in Nigeria. According to the World Prison Brief, the prison population rate per 100,000 population increased from 27 in 2008 to 37 in 2018. The majority (72%) of the prison population are pre-trial detainees [8]. Because of lack of testing and reporting, data on the COVID-19 disease burden in Nigerian prisons is sparse. At the time of writing this report, we have not found any outbreak data for prisons either on the Nigeria Center for Disease Control COVID-19 dashboard or reported in any medical or public health publication. Recently, a newspaper article reported that 17 inmates at a prison in Bauchi State were infected with COVID-19 and subsequently isolated [9]. Additional information, such as hospitalizations or deaths within this population was not reported. Prisoners form a bridge with the local community; therefore, testing, timely case identification, and quarantine or isolation are important steps to reduce disease transmission at the community level [10]. In a recent report from United States Centers for Disease Control and Prevention (CDC), early testing led to the identification of disease in asymptomatic and pre-symptomatic prisoners. The CDC further recommended that serial testing should be carried out for close contacts of positive cases to identify new cases. COVID-19 positive individuals should be either quarantined or isolated [11].

A centralized COVID-19 data collection system for the Nigerian Correctional Service would enhance the understanding of disease spread and could be used to design evidenced based polices to control the spread of disease within prisons and from prisoners to the outside community. The data collected could be used to measure the epidemiological burden of COVID-19 and monitoring response efforts in a timely manner. The proposed database should include the total prison population and should record the number of prisoners who undergo testing, the number who test positive and negative, the number of hospitalizations, and number of deaths, along with detailed demographic information i.e. age, gender, co-morbidities. Data should be stratified by State and correctional facility to enhance understanding of which States and facilities are most affected. Data should be integrated into public health monitoring at the national level and be accessible to those involved in COVID-19 prison research. This information could be used to mitigate future risks, identify occupational health deficits, and inform prison and national policies. In the United States, many prisoners have been diagnosed with COVID-19, but, through active surveillance and reporting, correctional facilities have been able to decrease the spread of the disease within the facilities [12-14]. We feel that strategies like those used in US correctional facilities, can be applied to facilities in other countries, such as Nigeria, to enhance their COVID-19 response.
